# Aberrant right subclavian artery re-routing for hybrid repair of proximal descending aortic aneurysm

**DOI:** 10.1186/s13019-018-0817-3

**Published:** 2018-12-20

**Authors:** Tae Yun Kim, Kyung Hwa Kim

**Affiliations:** 1 Department of Thoracic and cardiovascular surgery, Chonbuk National University Medical School, Chonbuk National University Hospital, 20 Geonji-Ro, Geumam-dong, Deokjin-gu, Jeonju, 54907 Republic of Korea; 2 Research Institute of Clinical Medicine of Chonbuk National University and Biomedical Research Institute of Chonbuk National University Hospital, 20 Geonji-Ro, Geumam-dong, Deokjin-gu, Jeonju, 54907 Republic of Korea

**Keywords:** Aberrant right subclavian artery, Proximal descending aortic aneurysm

## Abstract

**Background:**

An aberrant right subclavian artery (ARSA) is a relatively prevalent vascular anomaly. What is the most appropriate treatment for thoracic aortic aneurysm combined a non-aneurysmal change ARSA?

**Case presentation:**

A 52-year-old man was admitted to our institute due to a history of chronic cough, dysphagia and an abnormal chest radiographic finding. Because of his progressive symptoms and large fusiform thoracic aneurysm, we performed the hybrid repair for simultaneous relief of an ARSA causing dysphagia and thoracic aneurysm.

**Conclusion:**

In case without aneurysm of ARSA, especially in conjunction with approximate thoracic aneurysm, our approach is suitable because the revascularization using the right carotid to subclavian artery re-routing prior to endograft deployment is justified in order to preserve circulation of posterior brain, spinal cord, internal mammary artery and upper limb and to prevent large retrograde type II endoleaks, as well as simplicity and durability.

## Background

An aberrant right subclavian artery (ARSA) is a relatively prevalent vascular anomaly [1]. We considered several treatment options for throracic aortic aneurysm combined non-aneurysmal formation ARSA. For example, there were the isolated open surgery for thoracic aneurysm due to relatively young age, or selective thoracic endograft for simplicity, or occlusion the orifice of the aberrant right subclavian artery using an vascular plug and added a carotid-subclavian bypass / transposition via a supraclavicular incision. In the endovascular era, we presented the hybrid repair for simultaneous relief of an ARSA causing dysphagia and thoracic aneurysm.

## Case presentation

**Fig. 1:**
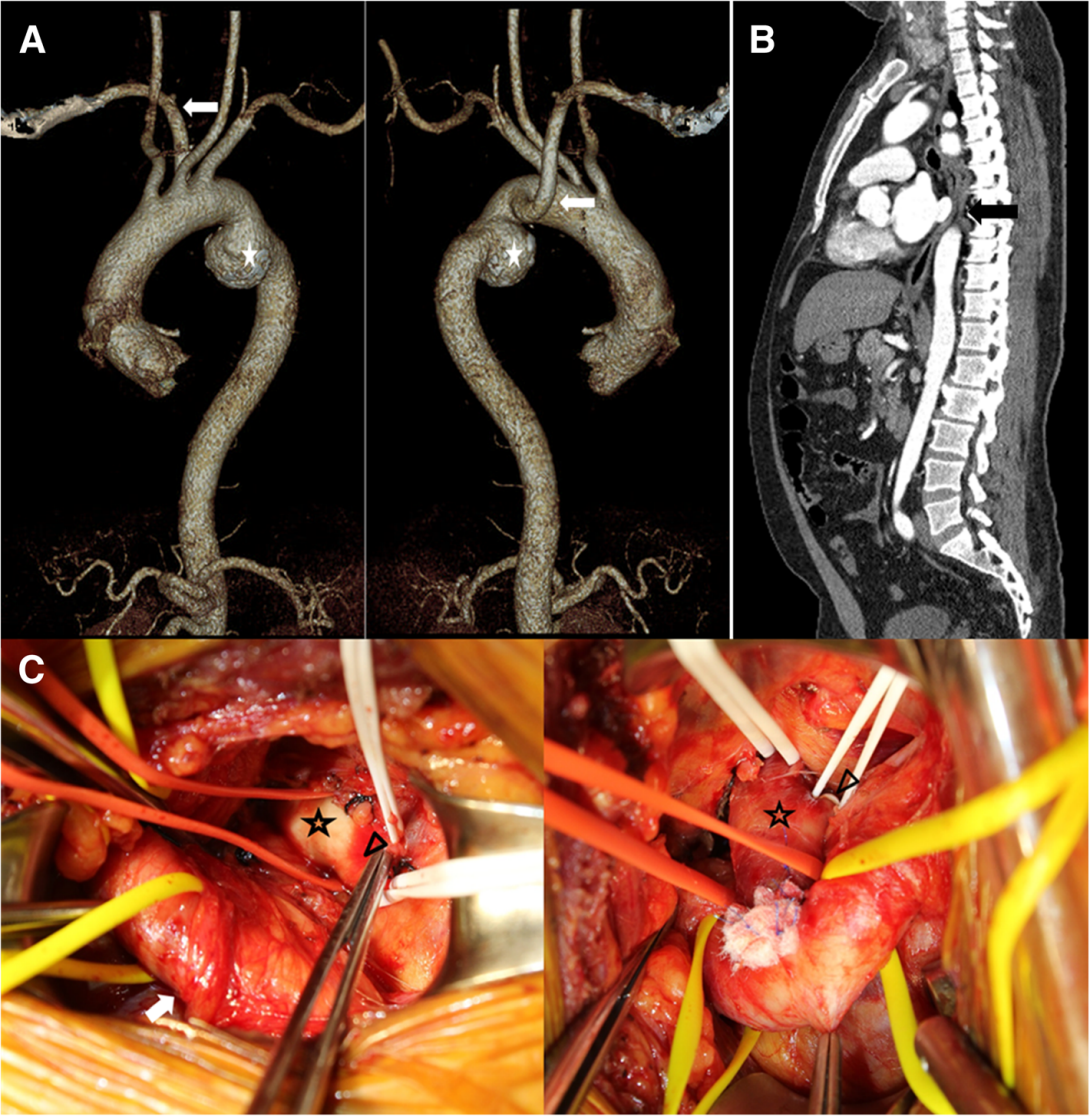
**a**-**b** Computed tomographic angiography showed a fusiform proximal descending aortic aneurysm (asterisk) distal to an ARSA (white arrow) with compressed esophagus (black arrow). **c** In operative field, ARSA (asterisk) to right carotid artery transposition (white arrow) with a ligation of the ARSA distal to the right vertebral (arrow head) and mammary arteries

**Fig. 2:**
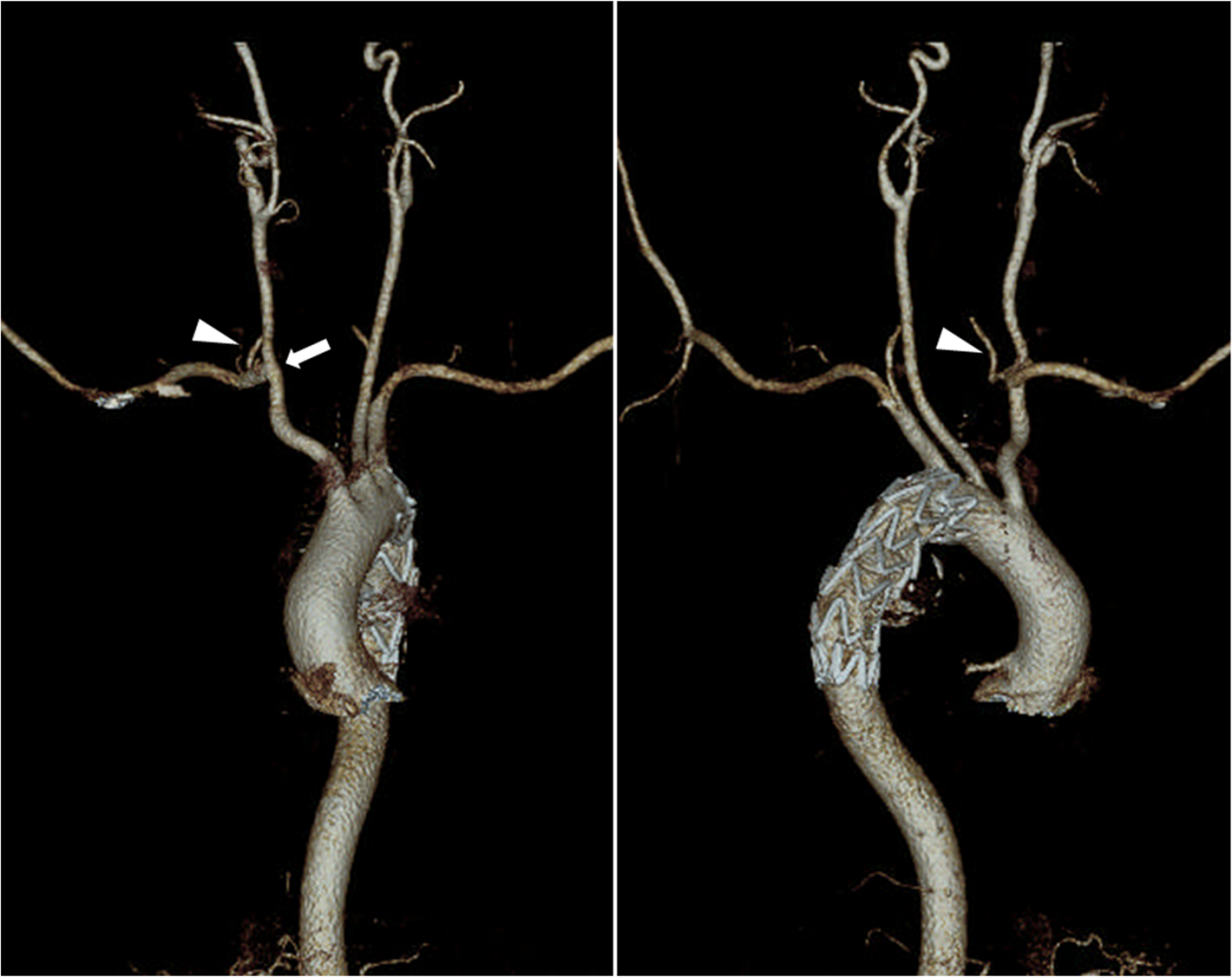
Computed tomographic angiography showing the result of hybrid treatment with transposition of right subclavian artery in the right common carotid artery (arrow) with intact right vertebral artery vertebral (arrow head) and thoracic endovascular aneurysm repair with complete occlusion of ARSA

A 52-year-old man was admitted to our institute due to a history of chronic cough, dysphagia and an abnormal chest radiographic finding. A chest x-ray and computed tomographic angiography scan (CTA) revealed an ARSA behind the esophagus with about 5.6-cm sized proximal descending aortic aneurysm (Fig. 1a). The esophagus was clearly compressed by the ARSA (Fig. 1b), likely causing the dysphagia. Both carotid arteries had a common origin. Because of his progressive symptoms and large fusiform thoracic aneurysm, we planned the hybrid repair for simultaneous relief of ARSA causing dysphagia and thoracic aneurysm. First, an ARSA to the right carotid artery transposition with a proximal ligation of the ARSA along distal to the right vertebral and mammary arteries was performed via the right supraclavicular incision (Fig. 1c). One hour later, we performed a thoracic endovascular aortic repair (TEVAR), deploying of a thoracic endovascular covered stent graft (Valiant™ thoracic stent graft with the Captivia™ delivery system) in the descending thoracic aorta with the coverage of the origin of the ARSA and the proximal descending thoracic aneurysm. The postoperative recovery was uneventful. The follow-up thoracic CTA revealed no endoleak, no graft migration, and complete exclusion of the ARSA and aneurysm. The right carotid to subclavian artery re-routing was showed to be excellent structural integrity and normal flow patterns with well-preserved right vertebral artery and right upper limb flow (Fig. 2). He was asymptomatic with complete resolution of his cough and dysphagia.

## Discussion and conclusion

An ARSA is the most common congenital abnormality of the aortic arch [2]. An ARSA does not usually cause symptoms and can be discovered incidentally during life, or may be an incidental finding at autopsy [1, 2]. Thoracic aneurysm combined with an ARSA require the specific therapy because both atherosclerosis with progressive aortic elongation and aneurysm formation of the ARSA. Also, the isolated ARSA can result in dysphagia or dyspnea due to compression of the esophagus or the trachea and with this, aneurysmal formation at this location can cause serious complications, such as rupture, dissection, compression of neighboring structures, and distal embolization. Treatment is recommended if the patient is symptomatic, has a Kommerell diverticulum > 3 cm in diameter, a symptomatic descending aortic aneurysm, or aneurysmal rupture [1, 3]. In our patient, the large-fusiform proximal descending aortic aneurysm located at Zone 3 and a symptomatic ARSA causing dysphagia convinced us to performed repair.

Conventional open repair for ARSA combined thoracic aneurysm is still associated with high mortality and morbidity, as well as neurological events [1, 2]. Therefore, nowadays, in order to provide a less invasive treatment, a hybrid approach consisting of subclavian-to-carotid transposition or bypass and TEVAR employing straight stent-graft is still advisable. We planned single-stage hybrid procedure. First, we performed right common carotid-to-right subclavian artery transposition along with ARSA proximal ligation. One hour later, we performed TEVAR. During TEVAR for combined aortic aneurysm, the patients with coverage of the ASA without revascularization are at a higher risk of neurological complications such as posterior stroke and ischemic spinal cord damage than patients with revascularization [3, 4, 5]. We think that the right carotid to subclavian artery re-routing revascularization is a better strategy than bypass surgery using the artificial graft in order to preserve circulation of posterior brain, spinal cord, internal mammary artery and upper limb and to prevent large retrograde type II endoleaks, as well as simplicity and durability. We confirmed the preservation of the right vertebral artery and right upper limb flow in follow-up CTA and no postoperative neurologic complication for 2 years.

## Abbreviations

ARSA: Aberrant right subclavian artery
CTA: Computed tomographic angiography scan
TEVAR: Thoracic endovascular aortic repair
